# The Impact of Education and Lifestyle Factors on Disability-Free Life Expectancy From Mid-Life to Older Age: A Multi-Cohort Study

**DOI:** 10.3389/ijph.2022.1605045

**Published:** 2022-08-12

**Authors:** Md. Mijanur Rahman, Carol Jagger, Lucy Leigh, Elizabeth Holliday, Emily Princehorn, Deb Loxton, Paul Kowal, John Beard, Julie Byles

**Affiliations:** ^1^ The Daffodil Centre, The University of Sydney and Cancer Council NSW, Sydney, NSW, Australia; ^2^ Population Health Sciences Institute, Newcastle University, Newcastle upon Tyne, United Kingdom; ^3^ Hunter Medical Research Institute, University of Newcastle, Newcastle, NSW, Australia; ^4^ School of Medicine and Public Health, The University of Newcastle, Newcastle, NSW, Australia; ^5^ World Health Organization (Switzerland), Geneva, Switzerland; ^6^ ARC Centre of Excellence in Population Ageing Research, University of New South Wales, Kensington, NSW, Australia

**Keywords:** women, disability-free life expectancy, low education, unhealthy lifestyle factors, multi-state Markov model

## Abstract

**Objectives:** Low education and unhealthy lifestyle factors such as obesity, smoking, and no exercise are modifiable risk factors for disability and premature mortality. We aimed to estimate the individual and joint impact of these factors on disability-free life expectancy (DFLE) and total life expectancy (TLE).

**Methods:** Data (*n* = 22,304) were from two birth cohorts (1921–26 and 1946–51) of the Australian Longitudinal Study on Women’s Health and linked National Death Index between 1996 and 2016. Discrete-time multi-state Markov models were used to assess the impact on DFLE and TLE.

**Results:** Compared to the most favourable combination of education and lifestyle factors, the least favourable combination (low education, obesity, current/past smoker, and no exercise) was associated with a loss of 5.0 years TLE, 95% confidence interval (95%CI): 3.2–6.8 and 6.4 years DFLE (95%CI: 4.8–7.8) at age 70 in the 1921–26 cohort. Corresponding losses in the 1946–51 cohort almost doubled (TLE: 11.0 years and DFLE: 13.0 years).

**Conclusion:** Individual or co-ocurrance of lifestyle risk factors were associated with a significant loss of DFLE, with a greater loss in low-educated women and those in the 1946–51 cohort.

## Introduction

Life expectancy has increased dramatically over the past 70 years in high-income countries including Australia, where a boy born today can expect to live over 81 years and a girl 85 years [1, 2]. However, the gain in life expectancy has not corresponded with similar increases in heathy or disability-free life expectancy (DFLE) for all [3–6]. A growing number of older people experience multiple chronic conditions and a high disability burden, posing huge challenges for healthcare systems and governments worldwide. The success of increased longevity could be diminished by negative health effects, such as multiple morbidities and progressive loss of physical, mental and cognitive capacity, with consequent impairment or disability [7]. Maximising healthy longevity—the time interval that an individual spends in good health and without limitations in functioning—is a major public health challenge and a policy imperative.

The World Health Organization has been advocating for the importance of healthy life expectancy (HLE) and DFLE over the last few decades and has recently declared the global public health goal “to live not just long but healthy lives” [8]. DFLE combines mortality and morbidity or disability data into a single index and quantifies the remaining years of life spent in a state of favourable or good health [9]. HLE and DFLE have been widely used to compare health in different populations, to explore inequalities in health between different sub-populations, and to monitor changes over time [3]. These measures are very useful for policy communication and public understanding of the health inequalities, targeting resources for health promotion, and planning and evaluating health, social and fiscal policy [9].

Numerous studies have demonstrated that unhealthy lifestyle factors, such as being physically inactive, obese, and a current/past smoker are associated with an increased risk of chronic disease and premature mortality [10–12]. A meta-analysis of 15 studies across 17 countries suggested that approximately 60% of premature death is preventable by reducing unhealthy lifestyle factors [13]. While several studies explored the impact of unhealthy lifestyle factors on total life expectancy (TLE) [14–16], few studies have explored chronic disease-free life [17, 18] and DFLE [3]. A US-based study reported that less than 12 years of schooling is associated with an increased prevalence of disability and decreased HLE or DFLE [19]. An international comparative study across 15 European countries reports that risk factors are more prevalent among low-educated women, and they had higher life with disability than those had high education, with a difference of 5.5 years [20].

A recent multi-cohort Australian study, focusing on the individual and combined effects of smoking and obesity, with adjustment for level of educational qualification, reported that obesity has the largest impact on mobility disability in women. Even highly educated, non-smoking obese women lived 5.1 fewer years free of mobility disability and an extra 3.5 years with mobility disability from age 65, with 1.3 years lower total life expectancy [21]. So far, no Australian study has compared the impact of the combination of two or more lifestyle risk factors and educational disparity on DFLE across different age cohorts.

Furthermore, there is a popular belief that “baby boomers,” a generation with distinct socio-economic characteristics and larger in size than earlier generations, are ageing in better health than previous generations. However, with the increased prevalence of unhealthy behavioural risk factors, recent cohorts are experiencing an all-cause mortality disadvantage in the US and Canada [22, 23]. No study has examined the joint impact of education and unhealthy lifestyle factors on years lived free of disability across two cohorts from two generations (baby boomers and the previous generation). With the opportunity to use over 20 years of longitudinal survey data from two birth cohorts (1921–26 and 1946–51) of the Australian Longitudinal Study on Women’s Health and linked National Death Index, this study aimed to.

1) Compare the prevalence of lifestyle risk factors between the two cohorts.
2) Estimate the DFLE, TLE, and the proportion of remaining disability-free life for different combinations of lifestyle factors and education across the two cohorts, and
3) Examine the impact of different combinations of lifestyle risk factors and low education on DFLE and TLE

## Methods

### Data Source and Sample

We used data from two birth cohorts (1921–26 and 1946–51) of the Australian Longitudinal Study on Women’s Health (ALSWH) and linked National Death Index over the period of 1996–2016. The ALSWH is a national population-based study that aims to examine factors associated with Australian women’s physical and emotional health across their lifespan. At baseline (1996), 12,432 women in the 1921–26 cohort and 13,714 women in the 1946–51 cohort were randomly selected from the Medicare Australia database, and respondents completed a self-reported postal questionnaire. Both cohorts roughly represent women of their respective age groups in the Australian national population. Women in the 1921–26 cohort were surveyed every 3 years until 2011 (six surveys) and thereafter on a six-monthly rolling basis (ten surveys until 2016). Women in the 1946–51 cohort were first followed up in 1998, and then surveyed every 3 years (eight surveys until 2016). Over the study period, the two cohorts together covered the life-course from mid-life to very old age (Table 1). Details about the ALSWH surveys and cohorts have previously been published elsewhere [24, 25].

**TABLE 1:** Study sample and exclusion criteria by cohorts (Australia, 2016).

Study sample	Birth cohorts			
	1946–51		1921–26	
Study period	1996 (baseline)	2016 (end)	1996 (baseline)	2016 (end)
Age	45–50	65–70	70–75	90–95
Participants	13,715	8186^d^	12,432	1362^d^
Excluded participants^a^	899^b^	**—**	2944^c^	**—**
Final Sample	12,816	**—**	9488	**—**

a Women who reported disability were excluded to remove the potential confounding of the results due to pre-existing disability and its association with reduced physical activity and higher body weights. A small proportion of underweight women (body mass index<18.5) were excluded as of its greater association with mortality. Furthermore, women who had missing information in covariate (after backfilling if available in subsequent surveys) were excluded to aid with the modelling requirements in the Interpolated Markov Chain (IMaCH) software.

b Includes 153 women who reported disability at baseline, 231 who had body mass index<18.5, and 514 had missing information in lifestyle factors and education.

c Includes 934 women who reported disability at baseline, 336 who had body mass index<18.5, and 1674 had missing information in lifestyle factors and education.

d Around 67% of women in the 1921–26 cohort and 6% of the 1946–51 cohort died by end of the study.

The final analytical sample included 9488 women from the 1921–26 cohort and 12,816 women from the 1946–51 cohort. Details about the excluded participants are provided in Table 1. Ethical approval was obtained from the Human Resources Ethics Committee (The University of Newcastle).

### Measure of Variables

Disability was defined as having a score below 40 in the Short Form-36 (SF-36) physical functioning domain and needing regular help with daily tasks because of long-term illness, disability or frailty. The SF-36 physical functioning scores were based on women’s responses (Yes, limited a lot; Yes, limited a little; and No not limited at all) at each survey across 10 items, including vigorous activities, moderate activities, lifting or carrying, climbing one or several flights of stairs, bending, bathing or dressing, and walking 100 m, half a kilometre or more than 1 km [26]. Initially, raw scores were calculated as the sum of the scale items, then transformed to a 1–100 scale, with higher scores indicating better health-related quality of life. Earlier studies reported that people with a physical functioning score below 40 are likely to have poor physical health and require help in managing moderate activities [27]. Needing regular assistance with daily tasks because of long-term illness, disability or frailty was a single question with the response options “yes” or “no”.

Education was categorised as low education (did not complete higher school certificate or Year 12) and high education (completed higher school certificate or higher qualifications).

Lifestyle factors included baseline smoking status, obesity, and level of exercise. For the current analysis, all variables were dichotomised to aid the modelling process. Obesity was defined as having a Body Mass Index (BMI) of ≥30 and “not obese” as a BMI between 18.5 and 30. Smoking status was categorised as “non-smoker” (those who never smoked) or “smoker” (current or past smoker). Participation in exercise was defined as those engaging either in vigorous exercise (such as jogging, squash, aerobics, and vigorous swimming) or non-vigorous activities (such as walking, gardening, swimming, and lawn bowls) for 20 min or more “at least once” in a typical week, or those who “never do” this level of exercise.

### Statistical Analysis

We summarised the two cohorts’ baseline demographic and lifestyle profiles using numbers and proportions and compared them by Chi-square tests. TLE, DFLE, and life expectancy with disability (DLE), were calculated using discrete-time multi-state models, implemented in the specialised health expectancy software “Integrated Markov Chain (IMaCH) version 0.99r23” [28]. IMaCH was designed to estimate transition probabilities from longitudinal survey data using a discrete-time embedded Markov chain. This technique models the transition between two transient states (no disability, disability) and an absorbing state (death) over the 21 years of the study period by partitioning the time intervals between successive follow-ups into shorter steps (i.e., 1 year to 1 month time interval), which approximate the underlying continuous-time process. The transition between the states may not occur between follow-ups if there is no change in health status. Implementing the model in IMach software requires a specific format of the input data which includes month and year of birth and death, month and day of each follow-up, and health status at each follow-up. This method does not require fixed follow-up intervals and can accommodate missed follow-up information using an interpolation method. Both time-invariant and time-variant covariates can be incorporated into the model, but the covariates must be dichotomous. Details about the methodology has been published elsewhere [28, 29], and the implementation of the model in IMaCH software is available at http://euroreves.ined.fr/imach/.

The transition probabilities between the states were modelled by multinomial logistic regression on age and available dummy covariates (education, smoking, obesity and exercise in the current study). TLE, DFLE and DLE were calculated separately for different combinations of lifestyle risk factors with low education and high education. The combinations included: 1) non-smoker, not obese, does exercise; 2) smoker, obese, no exercise; 3) smoker, not obese, does exercise; 4) non-smoker, not obese, no exercise; 5) non-smoker, obese, no exercise; 6) smoker, not obese, no exercise, 7) non-smoker, obese, does exercise and 8) smoker, obese, does exercise. As the IMaCh software can run a maximum of six combinations at a time, we reported six combinations of lifestyle factors in order of maximum frequency, and separately for low education and high education.

The impact of having different combinations of lifestyle factors and education was tested using the two-sample Z-test. Relative risk ratio (RRR) and 95% confidence intervals (CI) were estimated to assess the effect of covariates on transitioning between the three states. The 1921–26 cohort and 1946–51 cohort data were analysed separately to examine the impact of lifestyle factors on TLE, DFLE and DLE from midlife to very older age across the cohorts. Initial data preparation was conducted using SAS version 9.4 (SAS Institute, Inc., Cary, NC).

## Results

The 1921–26 cohort comprised 9488 women with a median age at baseline of 73 years (interquartile range (IQR): 71–74); the 1946–51 cohort consisted of 12,816 women with a median age at baseline of 48 years (IQR: 46–49). Participants in the 1946–51 cohort were more likely to have completed higher school certificate or higher education (50.3% versus (vs.) 29.1%), be obese (19.1% vs. 13.4%), be current or past smokers (46.0% vs. 36.5%), and to do exercise for 20 min or more at least once in a typical week (88.3% vs. 84.7%) than those in the 1921–26 cohort (Table 2).

**TABLE 2:** Distribution of participants by demographic, lifestyle factors and health conditions at baseline (Australia, 1996).

Baseline characteristics	Birth cohort: 1921–26 (*n* = 9488)%	Birth cohort: 1946–51 (*n* = 12,816)%
Area of residence		
Major cities	71.7	65.8
Inner/outer region or remote	28.3	34.2
Marital status		
Married/De Facto	58.1	83.3
Single/separated/divorced/widowed	41.9	16.7
Manage on available income		
Easy/not too bad	75.4	57.3
Difficult some/all the time or impossible	24.6	42.7
Comorbid condition		
0–1	43.0	68.0
≥2	57.0	32.0
Obesity		
Not obese	86.6	80.9
Obese (BMI ≥ 30)	13.4	19.1
Smoking status		
Never smoked	63.5	54.0
Smoker^a^	36.5	46.0
Education		
<Higher school certificate (12 years)	70.9	49.7
≥Higher school certificate	29.1	50.3
Level of exercise		
Never do exercise	15.3	11.7
At least once a week^b^	84.7	88.3

BMI, Body Mass Index, All the differences between the two cohorts are significant *p* < 0.01.

a Includes those who used to smoke or occasionally or regularly smoked.

b Includes those engaging either in vigorous exercise (such as jogging, squash, aerobics, and vigorous swimming) or non-vigorous activities (such as walking, gardening, swimming, and lawn bowls) for 20 min or more in a normal week for at least once.

Table 3 presents the estimated TLE, DFLE, and DLE for women in the 1921–26 cohort at age 70 for different combinations of baseline lifestyle factors, separately for women with low and high education. Compared to women with high education who were non-smokers, not obese and who did exercise, women with high education who did exercise but were obese and smoked could expect to live 2.5 (95%CI: 1.3–3.7) fewer years and 3.2 (95%CI: 2.3–4.1) fewer years of disability-free life. A substantial loss of DFLE (5.0 years, 95%CI: 3.2–6.8) and an increase in DLE (1.4 years, 95%CI: 1.0–1.8) were associated with being a smoker, obese and not doing exercise among women with low education.

**TABLE 3:** Total life expectancy (TLE), disability-free life expectancy (DFLE), and life with disability (DLE) at age 70, by education and lifestyle factors for birth cohort 1921–26 (Australia, 2016).

Education and lifestyle group^a^	TLE (95%CI)	p-value	DFLE (95%CI)	p-value	DLE (95%CI)	p-value
High education^b^						
Non-smoker, not obese, does exercise^c^ (*n* = 1279)	19.7 ((18.6–20.8)		17.5 (16.7–18.4)		2.1 (1.9–2.3)	
Non-smoker, obese, does exercise (*n* = 162)	18.4 (18.0–18.9)		15.4 (15.1–15.7)		3.0 (2.8–3.2)	
Smoker^d^, not obese, does exercise (*n* = 957)	18.5 (17.3–19.7)		16.0 (15.0–17.0)		2.5 (2.1–2.9)	
Non-smoker, not obese, no exercise (*n* = 126)	17.0 (16.6–17.4)		14.6 (14.2–15.0)		2.4 (2.0–2.8)	
Smoker, obese, does exercise (*n* = 102)	17.2 (16.7–17.7)		14.3 (14.0–14.7)		2.9 (2.6–3.1)	
Loss (−)/gain (+) for being obese	−1.3 (−2.4 to −0.1)	*p* < 0.03	−2.1 (−3.1 to −1.2)	*p* < 0.01	0.9 (0.6–1.2)	*p* = 0.10
Loss (−)/gain (+) for being a smoker	−1.2 (−2.8 to 0.4)	*p* = 0.14	−1.5 (−2.8 to −0.2)	*p* < 0.02	0.4 (0.1–0.9)	*p* = 0.08
Loss (−)/gain (+) for not doing exercise	−2.7 (−3.9 to −1.5)	*p* < 0.01	−2.9 (−3.9 to −2.0)	*p* < 0.01	0.3 (-0.2-0.8)	*p* = 0.01
Loss (−)/gain (+) for being a smoker and obese	−2.5 (−3.7 to −1.3)	*p* < 0.01	−3.2 (−4.1 to −2.3)	*p* < 0.01	0.8 (0.4–1.1)	*p* = 0.02
Low education						
Non-smoker, not obese, does exercise (*n* = 3199)	19.0 (18.1–19.9)		16.8 (16.1–17.5)		2.1 (1.9–2.3)	
Non-smoker, obese, does exercise (*n* = 508)	17.8 (17.4–18.2)		14.7 (14.5–14.9)		3.0 (2.8–3.2)	
Smoker, not obese, does exercise (*n* = 1678)	18.3 (17.1–19.5)		15.7 (15.1–16.3)		2.6 (2.1–3.1)	
Non-smoker, not obese, no exercise (*n* = 532)	18.4 (16.8–20.0)		14.7 (13.9–15.5)		3.6 (2.6–4.6)	
Smoker, obese, does exercise (*n* = 190)	17.0 (15.4–18.6)		13.4 (12.7–14.1)		3.6 (2.6–4.6)	
Smoker, obese, no exercise (*n* = 78)	14.7 (14.2–15.2)		11.2 (10.8–11.6)		3.5 (3.2–3.8)	
Loss (−)/gain (+) for being an obese	−1.2 (−2.2 to −0.2)	*p* < 0.01	−2.1 (−2.9 to −1.3)	*p* = 0.02	0.9 (0.6–1.2)	*p* = 0.07
Loss (−)/gain (+)for being a smoker	−0.7 (−2.2 to 0.8)	*p* = 0.36	−1.1 (−2.1 to −0.1)	*p* < 0.03	0.5 (-0.1-1.1)	*p* = 0.05
Loss (−)/gain (+)for not doing exercise	−0.6 (−2.4 to 1.2)	*p* < 0.52	−2.1 (−3.2 to −1.0)	*p* < 0.01	1.5 (0.5–2.5)	*p* = 0.03
Loss (−)/gain (+) for being a smoker and obese	−2.0 (−3.8 to −0.2)	*p* < 0.03	−3.4 (−4.4 to −2.4)	*p* < 0.01	1.5 (0.5–2.5)	*p* < 0.01
Loss (−)/gain (+) for being smoker, obese, and no exercise	−4.3 (−5.4 to −3.2)	*p* < 0.01	−5.6 (−6.4 to −4.8)	*p* < 0.01	1.4 (1.0–1.8)	*p* < 0.01
Loss(-)/gain(+) for being a low educated, obese, smoker and not doing exercise	−5.0 (−6.8 to −3.2)	*p* < 0.01	−6.4 (−7.8 to −4.8)	*p* < 0.01	1.4 (1.0–1.8)	*p* < 0.01

a Five common lifestyle combinations under high educated and low educated women are presented. One additional lifestyle profile (obese, smoker, and no exercise) was included in the low education group to compare the most favourable (high education, non-obese, non-smoker, and does exercise) and least favourable profile (low education, obese, smoker, and no exercise). However, this profile was not included in the high education group due to a low number of high educated women in both cohorts who were obsessed, smoked and did not exercise.

b Higher school certificate (12-years) or higher education.

c In a normal week, engaging either in vigorous (e.g., jogging, aerobics etc) or non-vigorous (walking, swimming etc) exercise lasting for 20 min at least once a week.

d Includes those who used to smoke or occasionally or regularly smoked.

The impact of the lifestyle factors on TLE and DFLE at age 70 was more pronounced in the 1946–51 cohort than in the 1921–26 cohort (Table 4). Co-occurrence of two unhealthy lifestyle factors, for example, smoking and obesity were associated with 7.4 (95%CI: 3.2–11.6) fewer years DFLE among women with high education and 7.3 (95%CI: 3.4–11.2) fewer years DFLE among women with low education compared to those with three healthy lifestyle factors (non-smoker, not obese and do exercise). A subtantial reduction in DFLE (11.1 years, 95%CI: 7.5–14.7) was observed for co-occurance of smoking, obesity and no exercise among women with low education, to compare women with low education who had three healthy lifestyle factors (non-smoker, not obese and do exercise).

**TABLE 4:** Total life expectancy (TLE), disability-free life expectancy (DFLE), and life with disability (DLE) at age 70, by education and lifestyle factors for the birth cohort 1946–51 (Australia, 2016).

Education and lifestyle group^a^	TLE (95%CI)	p-value	DFLE (95%CI)	p-value	DLE (95%CI)	p-value
High education^b^						
Non-smoker, not obese, does exercise^c^ (*n* = 2828)	22.5 (19.0–26.0)		22.4 (18.9–25.9)		0.1 (0.0–0.20)	
Non-smoker, obese, does exercise (*n* = 477)	19.2 (16.3–22.1)		18.8 (15.9–21.7)		0.4 (0.2–0.6)	
Smoker^d^, not obese, does exercise (*n* = 2168)	18.5 (15.6–21.4)		18.3 (15.4–21.2)		0.2 (0.1–0.3)	
Non-smoker, not obese, no exercise (*n* = 233)	19.1 (15.8–22.4)		18.9 (15.6–22.2)		0.2 (0.1–0.4)	
Smoker, obese, does exercise (*n* = 396)	15.5 (13.1–17.9)		15.0 (12.6–17.4)		0.6 (0.3–0.8)	
Loss (−)/gain (+) for being obese	−3.3 (−7.9 to 1.3)	*p* < 0.16	−3.6 (−8.2 to 1.0)	*p* = 0.12	0.3 (0.1–0.5)	*p* < 0.01
Loss (−)/gain (+) for being a smoker	−4.0 (−8.6 to 0.6)	*p* = 0.08	−4.1 (−8.7 to 0.5)	*p* = 0.08	0.1 (0.0–0.2)	*p* = 0.26
Loss (−)/gain (+) for no exercise	−3.4 (−8.3 to 1.5)	*p* = 0.17	−3.5 (−8.4 to 1.4)	*p* = 0.15	0.1 (−0.1–0.3)	*p* = 0.22
Loss (−)/gain (+) for being a smoker and obese	−7.0 (−11.2 to −2.8)	*p* < 0.01	−7.4 (−11.6 to −3.2)	*p* < 0.01	0.5 (0.2–0.7)	*p* < 0.01
Low education						
Non-smoker, not obese, does exercise (*n* = 2240)	20.7 (17.6–23.8)		20.5 (17.4–23.6)		0.2 (0.0–0.4)	
Non-smoker, obese, does exercise (*n* = 657)	17.4 (14.9–19.9)		16.7 (14.2–19.2)		0.7 (0.3–1.1)	
Smoker, not obese, does exercise (*n* = 2035)	16.8 (14.3–19.3)		16.5 (14.0–19.0)		0.3 (0.1–0.5)	
Non-smoker, not obese, no exercise (*n* = 281)	17.3 (14.4–20.2)		16.9 (14.0–19.8)		0.4 (0.2–0.7)	
Smoker, obese, does exercise (*n* = 520)	13.8 (1.211.4–16.2)		13.2 (10.8–15.6)		0.6 (0.2–1.0)	
Smoker, obese, no exercise (*n* = 132)	11.5 (9.5–13.5)		9.4 (7.6–11.2)		2.1 (0.9–3.3)	
Loss (−)/gain (+) for being an obese	−3.3 (−7.3 to 0.7)	*p* = 0.11	−3.8 (−7.8 to 0.2)	*p* = 0.06	0.5 (0.1–0.9)	*p* = 0.28
Loss (−)/gain (+) for being a smoker	−3.9 (−7.9 to 0.1)	*p* = 0.06	−4.0 (−8.0 to 0.0)	*p* = 0.05	0.1 (−0.2−0.4)	*p* = 0.21
Loss (−)/gain (+) for no exercise	−3.4 (−7.7 to 0.9)	*p* = 0.12	−3.6 (−7.9 to 0.7)	*p* < 0.10	0.2 (−0.1−0.5)	*p* < 0.01
Loss (−)/gain (+) for being a smoker and obese	−6.9 (−10.8 to -3.0)	*p* < 0.01	−7.3 (−11.2 to −3.4)	*p* < 0.01	0.4 (−0.1−0.8)	*p* = 0.02
Loss (−)/gain (+) for being a smoker, obese, and not doing exercise	−9.2 (−12.9 to −5.5)	*p* < 0.01	−11.1 (−14.7 to 7.5)	*p* < 0.01	1.9 (0.7–3.1)	*p* < 0.01
Loss (−)/gain (+) for being a low educated, obese, smoker and not doing exercise	−11.0 (−15.5 to −6.5)	*p* < 0.01	−13.0 (17.4 to −8.6)	*p* < 0.01	2.0 (0.8–3.2)	*p* < 0.01

a Five common lifestyle combinations under high educated and low educated women are presented. One additional lifestyle profile (obese, smoker, and no exercise) was included in the low education group to compare the most favourable (high education, non-obese, non-smoker, and does exercise) and least favourable profile (low education, obese, smoker, and no exercise). However, this profile was not included in the high education group due to a low number of high educated women in both cohorts who were obsessed, smoked and did not exercise.

b Higher school certificate (12-years) or higher education.

c In a normal week, engaging either in vigorous (e.g., jogging, aerobics etc) or non-vigorous (walking, swimming etc.) exercise lasting for 20 min at least once a week.

d ncludes those who used to smoke or occasionally or regularly smoked.

At age 70 in the 1921–26 cohort, the loss of TLE and DFLE for women with the least fvourable combination of risk factors (low education, obese, smoker, and no exercise) compared to the most favourable (high education, not obese, non-smoker, and do exercise) was 5.0 (95%CI: 3.2–6.8) years and 6.4 (95%CI: 4.8–7.8) years, respectively. The corresponding values were almost doubled in the 1946–51 cohort, with a loss of TLE of 11.0 (95%CI: 6.5–15.5) years and DFLE of 13.0 (95%CI: 8.6–17.4) years. In contrast to TLE and DFLE, DLE increased among women with the least favourable lifestyle factors, by 1.4 (95%CI: 1.0–1.8) years in the 1921–26 cohort and 2.0 (95%CI: 0.8–3.2) years in the 1946–51 cohort.

**FIGURE 1:**
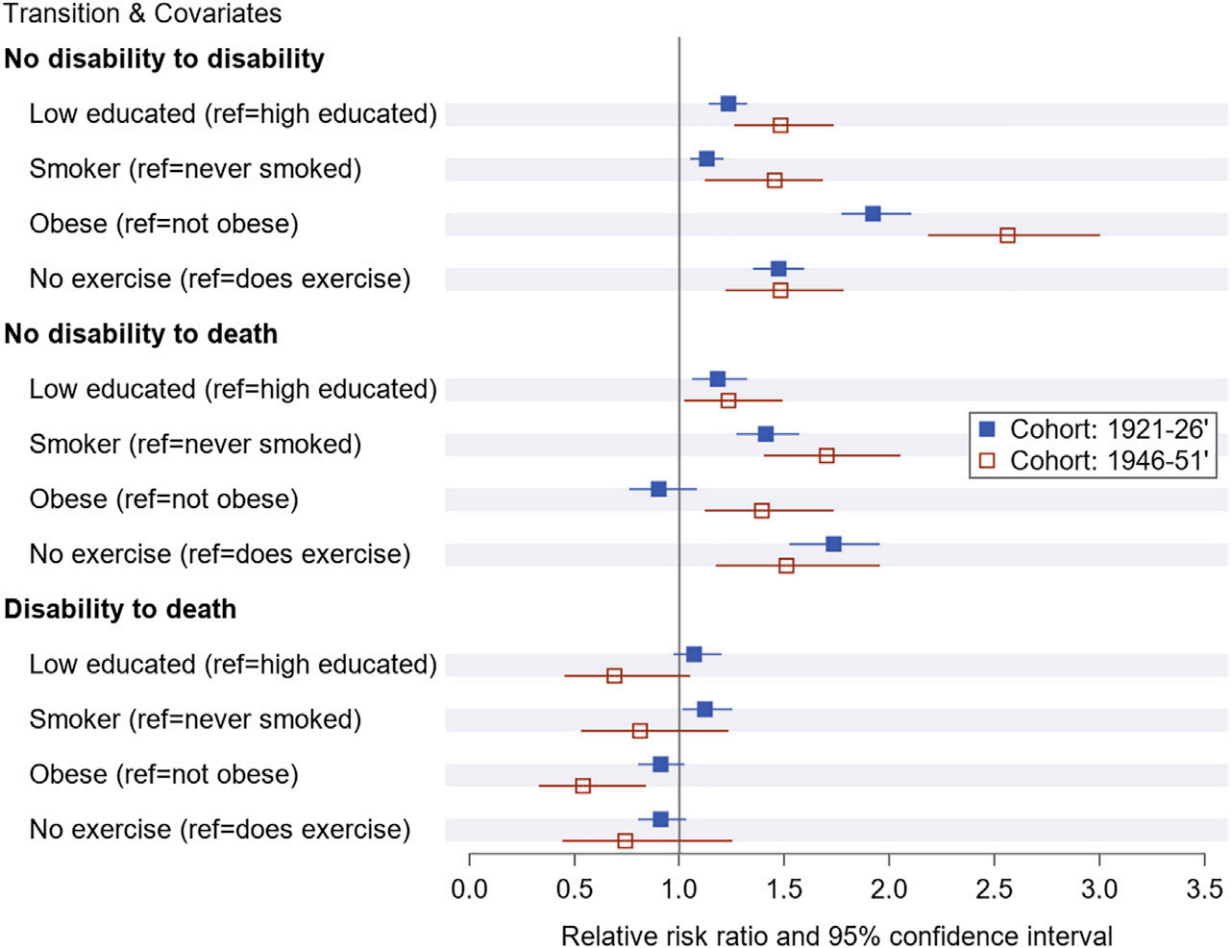
Relative risk ratio and 95% confidence for baseline education and lifestyle factor on transitioning to disability and death by cohorts over the study period from1996 to 2016 in Australia (Australia, 1996–2016).

The relative risk ratios (RRR) reveal that women with low education, who were obese, smoked, or did not exercise, had an increased risk of developing disability compared to their counterparts who were highly educated, not obese, non-smokers and who did exercise (Figure 1). For example, obese women in the 1946–51 cohort were 2.56 times more likely to develop disability than those who were not obese (RRR = 2.56, 95% CI = 2.18–3.0). Once women transitioned to a disability state, the effect of education and lifestyle factors were not significantly associated with the transition to death state at *p* < 0.05.

## Discussion

We estimated the impacts of low education and lifestyle risk factors on DFLE and TLE among two large cohorts of Australian women using longitudinal data from 1996 to 2016. The main finding is that low education and unhealthy lifestyle factors were associated with a substantial amount of loss of DFLE and TLE in both cohorts, with a greater loss of DFLE than TLE. At age 70 in the 1921–26 cohort, low educated women with three unhealthy lifestyle factors (obesity, smoking, and no exercise) had 6.4 years of fewer DFLE and 5.0 years of fewer TLE than high educated women with healthy lifestyle factors. The corresponding figures were almost double among women in the 1946–51 cohort (representing the leading edge of the post-war baby boom) with a loss of TLE of 11 years and DFLE of 13 years.

The greater loss in DFLE than in TLE suggests an increased disability-related burden on the healthcare system. We are not aware of other large-scale studies investigating the impact of the different combinations of lifestyle factors and education on DFLE or TLE. Our findings suggest that a reduction in population-level lifestyle risk factors and educational disparities could potentially increase DFLE and the proportion of remaining life free of disability [3, 30, 31]. This is potentially more important in the more recent cohort.

Although education is usually attained in early life, it often determines the subsequent socio-economic status and behavioural factors, with more highly educated people being more likely to exhibit healthy lifestyle factors [32, 33]. They are also more likely to have had adequate access to medical care, nutrition, and less stress in their lives–altogether affecting their life expectancy, as well as their ability to do exercise, their body size, and the likelihood that they will develop a chronic disease. Research suggests that educational attainment is one of the critical factors shaping the risk of disability and mortality, as chronic diseases are primarily associated with lifestyle factors [34, 35]. In line with this, our study demonstrated that being less educated, coupled with unhealthy lifestyle factors, was associated with shorter DFLE and increased DLE compared to having higher education and more healthy lifestyle factors [36]. A recent study in 15 European countries reports that several risk factors (including bodyweight and smoking) contribute educational inequalities in DFLE [37]. We observed that the educational disparity in HLE was wider in the 1921–26 cohort than in the 1946–51 cohort, given women in the former cohort had quite different educational opportunities (<30% higher school certificate) compared to those in the later cohort (almost 50%).

Our findings of an association between DFLE or TLE and unhealthy lifestyle factors were broadly consistent with previous studies [3, 14, 15, 30, 31, 38]. However, there are variations across these studies in terms of age of study participants, settings, lifestyle risk factors, follow-up period, analytical methods, and outcomes which may explain slight variations compared to our findings. Evidence from two large longitudinal studies on ageing revealed that increasing numbers of lifestyle risk factors were associated with lower health expectancy in the United States and England. For example, women with two or more behavioural risk factors had almost 7 years lower DFLE in the United States and nearly 6 years lower DFLE in England compared to those with no behavioural risk factors at age 70 [3]. An earlier study in the current older cohort reported that overweight/obese women had significantly lower healthy years of life and more years of unhealthy life at age 75 compared to those of normal weight [38].

We found that low education, smoking, obesity and not exercising were independently associated with an increased relative risk of transitioning to disability or to death among women in both cohorts. Research suggests that people with lower education levels are likely to have unhealthy lifestyle factors, which increase their likelihood of experiencing negative health outcomes [20, 36]. Smoking, a prevalent modifiable factor among low-educated people [39], is associated with a significant loss of years of life [21]. A substantial body of literature has demonstrated that unhealthy lifestyle factors, for example obesity is associated with increased risk of morbidity, mortality, and reduced life expectancy with increased disability compared to those maintaining healthy weight [38, 40, 41]. Similarly, physical inactivity, which is a leading cause of obesity, is also recognised as one of the critical risk factors of chronic health conditions, functional disability, mortality and reduction of healthy life expectancy [42–44]. However, obesity was not significantly associated with death among women in the 1921–26 cohort. This might partly be because overweight in older age is slightly protective against mortality, particularly for women in poor health [38].

Overall, the current study reveals a noticeably high negative association of lifestyle risk factors (obesity, smoking and no physical exercise) on DFLE among women in the 1946–51 cohort between their ages of 45 and 70 years. A high negative impact of these three lifestyle risk factors was also reported on HLE between mid to early older age (age 50–75) participants in a multi-cohort study across four European countries (UK, Sweden, France and Finland), suggesting that women with at least two of the unhealthy lifestyle risk factors had on average 7 years lower HLE than those with no risk factors [31]. Our findings do not provide evidence that the 1946–51 cohort (first cohort baby boomers generation) are ageing in better health than previous generations [45] and emphasise that such gains are dependent on educational status and health behaviours. However, it is important to note that women in the older generation (1921–26) are likely to be a healthier group of 70 year old people (i.e., healthier survivors) than the younger generation (1946–51) when they get to 70. A recent study in the United States and Canada also suggested that baby boomers had a higher susceptibility to behavioural causes of death [22]. Another US-based study among cancer survivors reported an expansion of life spent in disability among a more recent birth cohort (born 1948–57) compared to the older birth cohort (1918–27) [46]. Researchers in Switzerland reported that most health indicators did not suggest any indication of compression of morbidity in the baby boomer cohort [47]. To our knowledge, no other study is available regarding the comparison of health between cohorts of the general population from two generations, including baby boomers and the preceeding generation [48].

A major strength of our study is the use of data from two large representative cohorts of women from two generations with different life-course spectrums. When combining the age of women in the two cohorts over the 2 decades of follow-up, it covers women’s health and lifestyle trajectories from mid to older age which is so far the largest age coverage longitudinal cohort study in Australia and even elsewhere. This allowed us to examine the differential impacts of lifestyle risk factors not only in mid-life to old age but also across two generations. The multiple follow-ups over a long time provided sufficient transitions between being free of disability, life with disability and death to estimate the incidence of disability, recovery, and state-specific mortality rates. Furthermore, the use of Markov multi-state method for computing the DFLE based on the incidence of disability provides an understanding about the impact of the lifestyle risk factors on the individual life cycle as well as the implications of current conditions for future population-level changes [49].

The findings of the current study should be considered in light of some limitations. Our analysis is based on women only and therefore may not be generalisable to men. Previous studies demonstrate that women live longer than men but with shorter DFLE, suggesting that women have lower proportions of remaining life free of disability than men [50]. Furthermore, the impact of lifestyle factors on DFLE differs by gender; for example, smoking has a greater impact among men and physical inactivity has a greater impact among women [51]. The 3-year intervals between the follow-ups could miss transitions from disability-free to disability or recovery from disability. A recent multi-cohort study including the 1921–26 cohort of the current study reported that the impact of obesity and smoking on DFLE with follow-up at three yearly intervals did change when reanalysing with 1 year follow-up intervals [52].

The DFLE in the 1946–51 cohort may be overestimated in our study, because the incidence of disability in this cohort was relatively low, with the impact of lifestyle risk factors yet to be exhibited, given their age. If the increased midlife obesity among these women continues in older age, we expect a further shorter DFLE. However, after age 65, body weight starts to decline as a natural part of the ageing process, so we expect a lower level of obesity in this cohort when they turn 70. Additionally, the analytical approach in our study used dichotomous covariates and so we were unable to estimate the impact of different categories of covariates, for example, we did not consider overweight (BMI: 25–30) as a separate category which may also negative health outcomes.

The estimate of DFLE/TLE at age 70, for the 1946–51 cohort came from modelling the data at earlier ages (between age 45 and 70) and the lifestyle factors were measured at baseline when their age was 45–50. Whereas, for the 1921–26 cohort, the lifestyle factors were measured immediately at the start of the period when they were aged 70–75. Exclusion of women from the analysis due to missing covariate information may have implications for our findings; for example, we may expect - an increase in the estimate of DFLE/TLE in both cohorts if they have healthier combinations of lifestyle risk factors and higher education - and a reduction if they have unhealthier combinations of lifestyle risk factors and lower education. Furthermore, lifestyle factor often acts as a mediator of the association between education and health outcomes. However, no mediation analysis was performed in the current study, only the directed effects of education and lifestyle factors were considered.

### Conclusion

This study has shown that individual or co-occurrence of lifestyle risk factors (obesity, smoking and no physical exercise) substantially reduced the years of life without disability in both low- and high-educated women. While accounting for educational differences, the reduction of years of life without disability further worsened among low educated women, with greater reduction in the 1946–51 cohort than in the 1921–26 cohort. These findings suggests that there would be an increased burden of disability in the foreseeable future with the ageing of the recent cohort unless targeted action is taken to mitigate the impact of obesity and smoking. Our findings have implications in undertaking targeted health promotion interventions to adopt healthy lifestyle behaviour before reaching older age. Finally, investing in education, eradicating obesity and smoking, and increasing physical activity are paramount for healthy longevity in the population.
